# A Nomogram Modeling ^11^C-MET PET/CT and Clinical Features in Glioma Helps Predict IDH Mutation

**DOI:** 10.3389/fonc.2020.01200

**Published:** 2020-07-24

**Authors:** Weiyan Zhou, Zhirui Zhou, Jianbo Wen, Fang Xie, Yuhua Zhu, Zhengwei Zhang, Jianfei Xiao, Yijing Chen, Ming Li, Yihui Guan, Tao Hua

**Affiliations:** ^1^PET Center, Huashan Hospital, Fudan University, Shanghai, China; ^2^Department of Radiotherapy, Huashan Hospital, Fudan University, Shanghai, China; ^3^Department of Radiology, Huashan Hospital, Fudan University, Shanghai, China

**Keywords:** methionine, PET, nomogram, gliomas, isocitrate dehydrogenase mutation

## Abstract

**Purpose:** We developed a ^11^C-Methionine positron emission tomography/computed tomography (^11^C-MET PET/CT)-based nomogram model that uses easy-accessible imaging and clinical features to achieve reliable non-invasive isocitrate dehydrogenase (IDH)-mutant prediction with strong clinical translational capability.

**Methods:** One hundred and ten patients with pathologically proven glioma who underwent pretreatment ^11^C-MET PET/CT were retrospectively reviewed. IDH genotype was determined by IDH1 R132H immunohistochemistry staining. Maximum, mean and peak tumor-to-normal brain tissue (TNRmax, TNRmean, TNRpeak), metabolic tumor volume (MTV), total lesion methionine uptake (TLMU), and standard deviation of SUV (SUV_SD_) of the lesions on MET PET images were obtained via a dedicated workstation (Siemens. syngo.via). Univariate and multivariate logistic regression models were used to identify the predictive factors for IDH mutation. Nomogram and calibration plots were further performed.

**Results:** In the entire population, TNRmean, TNRmax, TNRpeak, and SUV_SD_ of IDH-mutant glioma patients were significantly lower than these values of IDH wildtype. Receiver operating characteristic (ROC) analysis suggested SUV_SD_ had the best performance for IDH-mutant discrimination (AUC = 0.731, cut-off ≤ 0.29, *p* < 0.001). All pairs of the ^11^C-MET PET metrics showed linear associations by Pearson correlation coefficients between 0.228 and 0.986. Multivariate analyses demonstrated that SUV_SD_ (>0.29 vs. ≤ 0.29 OR: 0.053, *p* = 0.010), dichotomized brain midline structure involvement (no vs. yes OR: 26.52, *p* = 0.000) and age (≤ 45 vs. >45 years OR: 3.23, *p* = 0.023), were associated with a higher incidence of IDH mutation. The nomogram modeling showed good discrimination, with a C-statistics of 0.866 (95% CI: 0.796–0.937) and was well-calibrated.

**Conclusions:** ^11^C-Methionine PET/CT imaging features (SUV_SD_ and the involvement of brain midline structure) can be conveniently used to facilitate the pre-operative prediction of IDH genotype. The nomogram model based on ^11^C-Methionine PET/CT and clinical age features might be clinically useful in non-invasive IDH mutation status prediction for untreated glioma patients.

## Introduction

Gliomas are the most prevalent malignant primary tumors of the brain. Over the past years, isocitrate dehydrogenase enzyme (IDH) mutations have been proven to be an inciting event in gliomagenesis, which made a great difference in the molecular and genetic route of oncogenic progression and clinical outcome (1). IDH mutations were identified in low grade glioma (LGG) and secondary glioblastoma multiforme (GBM) with a high percentage but in primary GBM with a much lower percentage (2). Glioma patients with IDH mutation had been prone to significantly better progression-free survival than those IDH wildtype counterparts, irrespective of grade or received treatments (3). Thereafter, some IDH wildtype LGGs can be as aggressive and have prognoses that are quite similar to GBMs (4). The gold standard of IDH mutations detection relies on immunohistochemistry or genetic sequencing of the surgical specimens. Given the inherent risk of surgery or biopsy, substantial research efforts have focused on the pre-operative non-invasive prediction of IDH mutational status in gliomas.

In 2016, the World Health Organization (WHO) updated the classification criteria for central nervous system tumors, in which IDH mutation and 1p/19q codeletion made a significant difference in the latest classification of glioma (5). The amino acid PET imaging has become increasingly important in evaluating the atypical non-enhancing gliomas as well as the differentiating tumor progression from treatment-related changes (6). Response Assessment in Neuro-Oncology (RANO) working group proposed that amino acid positron emission tomography (PET) imaging should be used in all aspects of glioma management combined with magnetic resonance imaging (MRI). L-[methyl-11C]methionine (^11^C-MET) PET imaging has been widely used in glioma grading, differential diagnosis, tumor scope definition, brain biopsy site determination, radiotherapy planning, prognostication, and treatment monitoring (7–12).

Radiomics analysis from multimodality MRI or FDG PET images have been reported to be sufficient for IDH prediction (13). A recent study (14) by Maldjian et al. evaluated the usefulness of a non-invasive, only T2 weighted MRI based deep-learning method for the determination of IDH status. The results are inspiring since T2-weighted MR imaging is widely available and routinely performed in the assessment of gliomas. Some studies have explored the relationship between amino acid uptake characteristics of gliomas and IDH mutation status (15–19). We aimed to develop a novel and convenient statistical model that combines PET features and clinical factors for an IDH-predictive signature. Nomogram is a prediction tool that creates a simple pictorial representation of a statistical prediction model that generates a probability of a clinical event and aid in clinical decision-making (20, 21). It is “a form of line chart showing scales of the variables involved in a particular formula in a way that corresponding values for each variable lie in a straight line intersecting all the scales.”(22) Therefore we tried to establish a MET PET/CT-based nomogram model that uses easy-accessible imaging metrics and clinical features to add reliable predictive information for IDH mutational status in patients with gliomas.

## Materials and Methods

### Study Population

We conducted a retrospective study of patients with histologically proven diffuse glioma who underwent ^11^C-MET PET/CT between February 2012 and November 2017 at a single center. Inclusion criteria: (1) all patients were confirmed to have glioma histological diagnosis and IDH1 R132H immunohistochemistrical staining results. (2) PET images for every patient were of good quality with no obvious artifacts. Exclusion criteria: (i) patients who received treatment by radiotherapy, chemotherapy, or chemoradiotherapy before PET imaging. (ii) glioma patients with no precise histological grading or IDH1R132H staining results. (iii) poor image quality with artifacts affecting the semi-quantitative analysis. (iv) hypo- or iso-metabolism of ^11^C-MET compared to the background which is not applicable for threshold-based tumor volume delineation procedures. Moreover, the interval between PET imaging and subsequent tumor resection or biopsy was no more than 100 days for grade II or III gliomas and no more than 30 days for grade IV glioblastomas. A total of 110 cases were eligible for inclusion.

### ^11^C-MET PET/CT Imaging Protocol

^11^C-MET was synthesized by the GE Healthcare-Tracerlab-FXc ^11^C radiolabelling module semi-automatically. ^11^C-CO_2_ was produced by SIEMENS RDS III cyclotron, and homo-cysteine was used as the precursor. The radiochemical purity of the obtained sterile product was higher than 95%. All patients had fasted for at least 4 h before imaging. At 10–15 min after an intravenous bonus injection of ^11^C-MET (370–550 MBq), a static PET scan was subsequently collected for 20 min with a Siemens Biograph 64 HD PET/CT (Siemens, Erlangen, Germany) in 3-dimensional (3D) mode. PET images were reconstructed using the filter back projection (FBP) with Gaussian filter (FWHM 3.5 mm) and a 256^*^256 matrix, providing 64 contiguous transaxial slices of 5 mm-thick spacing. Attenuation correction was performed using a low-dose CT (150 mAs, 120 kV, Acq. 64^*^0.6 mm) before the emission scan.

### ^11^C-MET PET/CT Data Analysis

All PET/CT images were analyzed using a dedicated workstation (Siemens.syngo.via). Semi-quantitative analysis of tumor metabolic activity was obtained using SUV normalized to body weight. All parameters were assessed in 3-dimensional volumes. Mean standardized uptake values (SUVmean) of the normal contralateral frontal cortex were calculated as references. A predefined threshold method at 1.3-times of the corresponding reference SUVmean (23, 24) was applied. The brain MRI of patients were reviewed initially to locate the possible tumor region. A VOI isocontour of the tumor region were applied, semi-quantitative PET imaging analysis were carried out after the lesion delineation procedures. The above-mentioned procedures were carried out by two experienced nuclear medicine physicians separately to double confirm the correct inclusion and reproducible parameters measurements of the glioma lesion. For those multifocal glioma patients in our group, the specific surgical resected or biopsied lesion for pathological examination were included in our research in order to avoid bias. Each VOI generated a maximum of SUV (SUVmax), a mean SUV (SUVmean), a peak SUV (SUVpeak), a standard deviation of SUVmean (SUV_SD_), a metabolic tumor volume (MTV) and a total lesion methionine uptake (TLMU). The total lesion methionine uptake (TLMU) was defined as the MTV multiplied by the SUVmean within the tumor boundary. SUVpeak was the highest mean SUV from a fixed 1-cm^3^ spherical volume centered over the highest metabolic part of the tumor. The lesion SUV/normal contralateral cortical SUVmean was defined as the tumor-to-normal brain tissue ratio (TNR) of ^11^C-MET uptake.

Physicians would examine the interested glioma lesions to decide whether brain midline structure were involved or not, mainly taking MET PET images for reference. The brain midline structures included corpus calloum, cingulate gyrus, thalamus, third ventricle and brain stem. As illustrated above, two physicians performed VOI delineation for each included patient to confirm the brain midline structure involvement status and to obtain two sets of MET metric features. In order to build a relatively stable integrated ^11^C-MET PET/CT metrics-based model, we evaluated the inter-observer agreement indices for those obtained results.

### Neuropathologic Analyses

Histological specimens were obtained by surgery or stereotactic brain biopsy. H&E staining and immunohistochemical analysis were performed by an experienced neuropathologist according to the current WHO guidelines. IDH status of the surgical samples was identified with an antibody to the IDH1 (R132H) mutation by immunohistochemical staining.

### Establishment of a MET PET-Based Nomogram and Validation of the Model Performance

Participant's age, gender and brain midline structure involvement were used as potential predictors, together with those MET-PET metrics, to perform the univariate logistic regression analysis for developing a prediction model of IDH mutation. Those MET-PET metrics share a deep homology, so we first evaluated their correlations to avoid overfitting in the nomogram model building. After that, the MET PET metrics-based nomogram was then designed based on a multivariable logistic analysis results in the whole group with the aim of providing the clinician with a quantitative tool used in the prediction of IDH mutation status. The nomogram model validation involved the quantitative assessment of the nomogram's accuracy in IDH mutation prediction by use of Harrell's concordance index (C-statistic) and calibration curve. The corrected C-index, which is used to quantify the level of concordance between predicted probabilities and actual chance, was measured to predict the accuracy (discrimination) of the nomogram (20, 25). A relatively corrected C-index could be calculated after bootstrap analyses using 1,000 resamples. The calibration curve was used to estimate how closely the modeled nomogram estimated the risk relative to the actual risk of IDH status (mutant or wildtype), accompanied by the Hosmer-Lemeshow test (26).

### Statistical Analysis

All continuous variables are expressed as mean ± standard deviation or median and range. Categorical variables are expressed as percentages. For continuous variables, an independent sample *t*-test was used to compare the two groups, while the chi-square test was applied to calculate *P*-values for categorical variables. Inter-observer agreements on ^11^C-MET PET metrics and dichotomized location results were assessed with interclass correlation coefficients (ICC) and Cohen's kappa coefficient analysis, respectively, defined as poor (<0.2), fair (0.21–0.4), moderate (0.41–0.6), good (0.61–0.8), and very good (0.8–1.0). All PET activity measuring indices were compared with each other using scatter plots and Pearson correlation coefficients. Receiver operating characteristic (ROC) analysis was performed to calculate the area under the ROC curve (AUC) for each PET semi-quantitative parameters. The Delong test was used in the comparison of ROC curves. The AUC of ROC curves analysis and the Delong test were performed by using MedCalc for windows (version11.3.3.0, MedCalc software, Mariakierke, Belgium). Univariate and multivariate logistic regression models were used to identify the predictive factors for an IDH mutation. A nomogram was formulated based on the results of multivariate logistic regression analysis and by using the rms package of R, version 3.6.1 (http://www.r-project.org/). The predictive performance of the nomogram was measured by concordance index (C-Statistics) and calibration with 1000 bootstrap samples to decrease the overfit bias. All other statistical analysis was performed using the Prism Software version 8.0 (GraphPad, San Diego. CA). In all analyses, *P* < 0.05 was considered to indicate statistical significance.

## Results

### Patient Demographics

The demographic data of the patients included in this study are listed in Table 1. Of the 110 patients who were evaluated retrospectively, 67 (59.32%) were male, and 43 (40.68%) were female, with a mean age of 45.5 years (range 10–71). The majority of patients (80/110) underwent tumor resection. The post-surgical histological examination demonstrated 59 grade II diffuse glioma, 32 grade III anaplastic tumors, and 19 grade IV glioblastomas, among which 61 patients confirmed IDH-wildtype while 49 patients were IDH-mutant. Patients with IDH-wildtype were more likely to present with lesions involving the midline structures, while there was no significant difference in gender distribution between these two groups (detailed in Table 2).

**Table 1:** Clinicopathological features of 110 patients.

**Characteristic**	**Numbers (Percentage %)**
**Age (median, range)**	45.5 years old (10–71)
**Gender**	
Male	67 (59.32%)
Female	43 (40.68%)
**Primary tumor location**	
Frontal	27 (24.54%)
Parietal	6 (5.45%)
Temporal	20 (18.18%)
Occipital	1 (0.91%)
Cerebellum	5 (4.55%)
Deep brain structure	14 (12.73%)
Multifocal	37 (33.64%)
**WHO grade classification**	
Grade II	59 (55.09%)
Grade III	32 (28.81%)
Grade IV	19 (16.10%)
**IDH status**	
Mutant (Grade II/III/IV)	42/7/0 (85.71%/14.29%/0.00%)
Wildtype (Grade II/III/IV)	17/25/19 (27.87%/40.98%/31.15%)
**Type of operation (surgery/stereotactic biopsy)**	
Grade II	47/12 (79.66%/20.34%)
Grade III	21/11 (65.63%/34.37%)
Grade IV	14/5 (73.68%/26.32%)

**Table 2:** Clinical features and ^11^C-MET PET metrics based on IDH-genotype.

**PET Metric**	**All patients (*n* = 110)**	**IDH-mutant (*n* = 49)**	**IDH-wildtype (*n* = 61)**	***P*-value^**a**^**
Age (mean ± SD)	45.08 ± 13.56	42.63 ± 10.6	47.05 ± 15.35	0.090
Age (≤ 45/>45 years)	55/55	31/18	24/37	0.013
Gender (M/F)	67/43	27/22	40/21	0.263
Midline Involvement (yes/no)	37/73	2/47	35/26	0.000
TNRmax	1.7719 ± 0.3038	1.6692 ± 0.2474	1.8544 ± 0.3211	0.001
TNRmean	3.2421 ± 1.3193	2.8277 ± 1.1741	3.5749 ± 1.3440	0.002
TNRpeak	2.8114 ± 1.1222	2.4739 ± 0.9704	3.0824 ± 1.1690	0.004
MTV	48.6750 ± 54.9081	44.9551 ± 53.7083	51.6631 ± 56.1161	0.525
TLMU	87.9881 ± 104.0785	68.5628 ± 86.9409	103.5920 ± 114.3505	0.071
SUV_SD_	0.3783 ± 0.2819	0.2551 ± 0.1781	0.4772 ± 0.3108	0.000

a comparison between IDH-mutant and IDH-wildtype.

### Inter-reader Agreement in ^11^C-MET PET Results

The dichotomized location results of the interested tumor lesion yielded very similar values for both readers, and accordingly, the inter-observer kappa was satisfactory (κ = 1.0, *p* < 0.0001). The ICC also showed perfect agreement for the five ^11^C-MET PET volume-based metrics (ICC > 0.95, *p* < 0.0001). Therefore, only the results of reader one were considered for further analysis. The absolute values for all ^11^C-MET PET metrics based on IDH-genotype were given in Table 2.

### Correlations of ^11^C-MET PET Metrics

All pairs of volume-based ^11^C-MET PET metrics showed a linear association, which was quantified by Pearson correlation coefficients. There were strong correlations between paired TNRs, i.e., TNRmax, TNRmean, and TNRpeak, and SUV_SD_ with r values ranging from 0.843 to 0.986 (*p* < 0.0001). The volume-related features, including MTV and TLMU, also correlated strongly with each other (*r* = 0.927, *p* < 0.0001). Intratumoral heterogeneity feature SUV_SD_ and TNRs demonstrated fair or moderate associations with MTV (*r* = 0.228–0.370, *p* < 0.05) and TLMU (*r* = 0.342–0.430, *p* < 0.0001) (detailed in Table 3 and Supplementary Figures 1A,B).

**Table 3:** Correlation of ^11^C-MET PET metrics.

**PET Metric**	**TNRmax**	**TNRmean**	**TNRpeak**	**MTV**	**TLMU**	**SUV_**SD**_**
TNRmax	1	0.843	0.986	0.364	0.419	0.861
TNRmean		1	0.872	0.242	0.342	0.855
TNRpeak			1	0.370	0.430	0.876
MTV				1	0.927	0.228
TLMU					1	0.401
SUV_SD_						1

### Pre-operative ^11^C-MET PET/CT ROC Analysis for IDH Mutation

As shown in Table 2, IDH-wildtype patients had significantly higher TNRmax, TNRmean, and TNRpeak values. Lower SUV_SD_ values were shown in IDH-mutant patients. Lower MTV and TLMU values were also observed in the IDH-mutant group, albeit not significantly so.

In the ROC analysis, the highest AUC of 0.731 (95%CI: 0.638–0.811) was reached by the SUV_SD_ value, with the best cut-off value at 0.29, a specificity of 60.66% and a sensitivity of 77.55%, followed by the TNRmax value with an AUC of 0.678, the best cut-off value at 2.99, a specificity of 59.02% and a sensitivity of 75.51%. Their optimal cutoff, AUC, sensitivity, specificity values, etc., for the abovementioned ^11^C-MET MET PET metrics were listed in Table 4. Their AUC curves were displayed in Supplementary Figure 2.

**Table 4:** The Performance of ^11^C-MET PET metrics and age feature for IDH-mutation prediction.

**PET metric**	**Cutoff**	**AUC (95% CI)**	**ACC**	**SEN**	**SPE**	**PPV**	**NPV**	**Youden-index**
SUV_SD_	≤ 0.29	0.731 (0.638–0.811)	68.18%	77.55%	60.66%	61.30%	77.10%	0.3821
TNRmax	≤ 2.9886	0.678 (0.582–0.764)	66.36%	75.51%	59.02%	59.70%	75.00%	0.3453
TNRmean	≤ 1.7051	0.679 (0.583–0.765)	65.46%	69.39%	62.30%	59.60%	71.70%	0.3168
TNRpeak	≤ 2.8191	0.660 (0.564-0.748)	65.45%	79.59%	54.10%	58.20%	76.70%	0.3369
Age	≤ 45	0.630 (0.533–0.720)	62.73%	65.31%	60.66%	57.10%	68.50%	0.2596
Midline involvement	yes	0.766 (0.676–0.842)	74.54%	95.92%	57.38%	64.40%	94.60%	0.5330

*CI, Confidence interval; AUC, Area under the receiver-operating characteristic curve; ACC, Accuracy; SEN, Sensitivity; SPE, Specitivity; PPV, Positive predictive value; NPV, Negative predictive value*.

Further pairwise comparisons of ROC curves confirmed that the AUC of SUV_SD_ differed significantly from any other MET PET metrics (*p* < 0.05, details in Supplementary Table 1).

### Predicting IDH-Mutant Gliomas and Construction of the Nomogram

In univariate analysis, the MET PET metrics including SUV_SD_, TNRmax, TNRpeak and TNRmean, except for MTV and TLMU, were significantly associated with IDH mutation (*p* < 0.05 for all the variables). Considering their collinearity and the AUC curve comparison results for the MET PET metrics, SUV_SD_ was selected as the only MET PET feature for further multivariate logistic regression analysis. In multivariate logistic regression analysis, the three factors, i.e., participant's age, the involvement of midline structure, and SUV_SD_, were found to be significant independent predictors. We demonstrated that SUV_SD_ (>0.29 vs. ≤ 0.29 OR: 0.053, *p* = 0.010), brain midline structure involvement (no vs. yes OR: 26.52, *p* = 0.000) and age (≤ 45 vs. >45 years OR: 3.23, *p* = 0.023), were associated with a higher incidence of IDH mutation (shown Table 5).

**Table 5:** Univariate and multivariate regression analyses for IDH mutation prediction.

		**Multivariate analysis**				
	**Univariate analysis**		**Model 1**		**Model 2**	
**Variable**	**OR (95% CI)**	***P***	**OR (95% CI)**	***P***	**OR (95% CI)**	***P***
Age (≤ 45 vs. >45)	2.655 (1.223–5.765)	0.014	3.232 (1.180–8.854)	0.023		
Gender (male vs. female)	0.644 (0.298–1.394)	0.264				
Midline structure Involvement (no vs. yes)	31.635 (7.035–142.248)	0.000	26.523 (5.547–126.831)	0.000	24.461 (5.305–112.789)	0.000
MTV	0.998 (0.991–1.005)	0.525				
TLMU	0.996 (0.992–1.001)	0.090				
TNRmean	0.093 (0.020–0.435)	0.003				
TNRmax	0.605 (0.427–0.857)	0.005				
TNRpeak	0.574 (0.385–0.856)	0.007				
SUV_SD_	0.02 (0.003–0.157)	0.000	0.053 (0.006–0.497)	0.010	0.048 (0.006–0.411)	0.006
C–index			0.866 (0.796–0.937)		0.843 (0.766–0.920)	

Age does not correlate with SUV_SD_ (*r* = 0.0370, *p* = 0.7010). Lesions involving brain midline structure (73 cases) showed higher SUV_SD_ (0.3147 ± 0.2501 vs. 0.5038 ± 0.3017, *p* = 0.007) compared to lesions without brain midline involvement (37 cases), but no age predominance (45.67 ± 12.10 vs. 43.92 ± 16.19, *p* = 0.5245) was observed for brain midline involvement (shown in Supplementary Figure 3).

**Figure 1:**
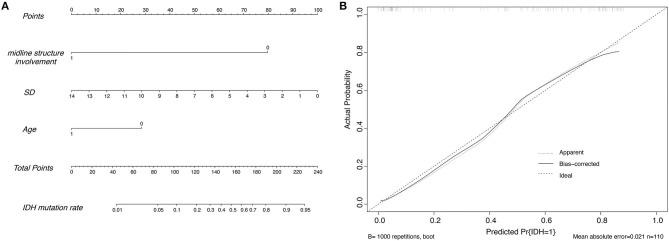
**(A)** The nomogram developed in the whole cohort using the SUV_SD_ metric, the age, and the brain midline structure involvement of the patients. 0 represents ≤ 45 years for age or without brain midline structure involvement and 1 represents >45 years for age or with brain midline involvement, respectively; SD = 100*SUV_SD_. **(B)** Calibration plots of the nomogram for predicting IDH mutation. The y-axis represents the actual probability, and the x-axis represents the predicted probability.

**Figure 2:**
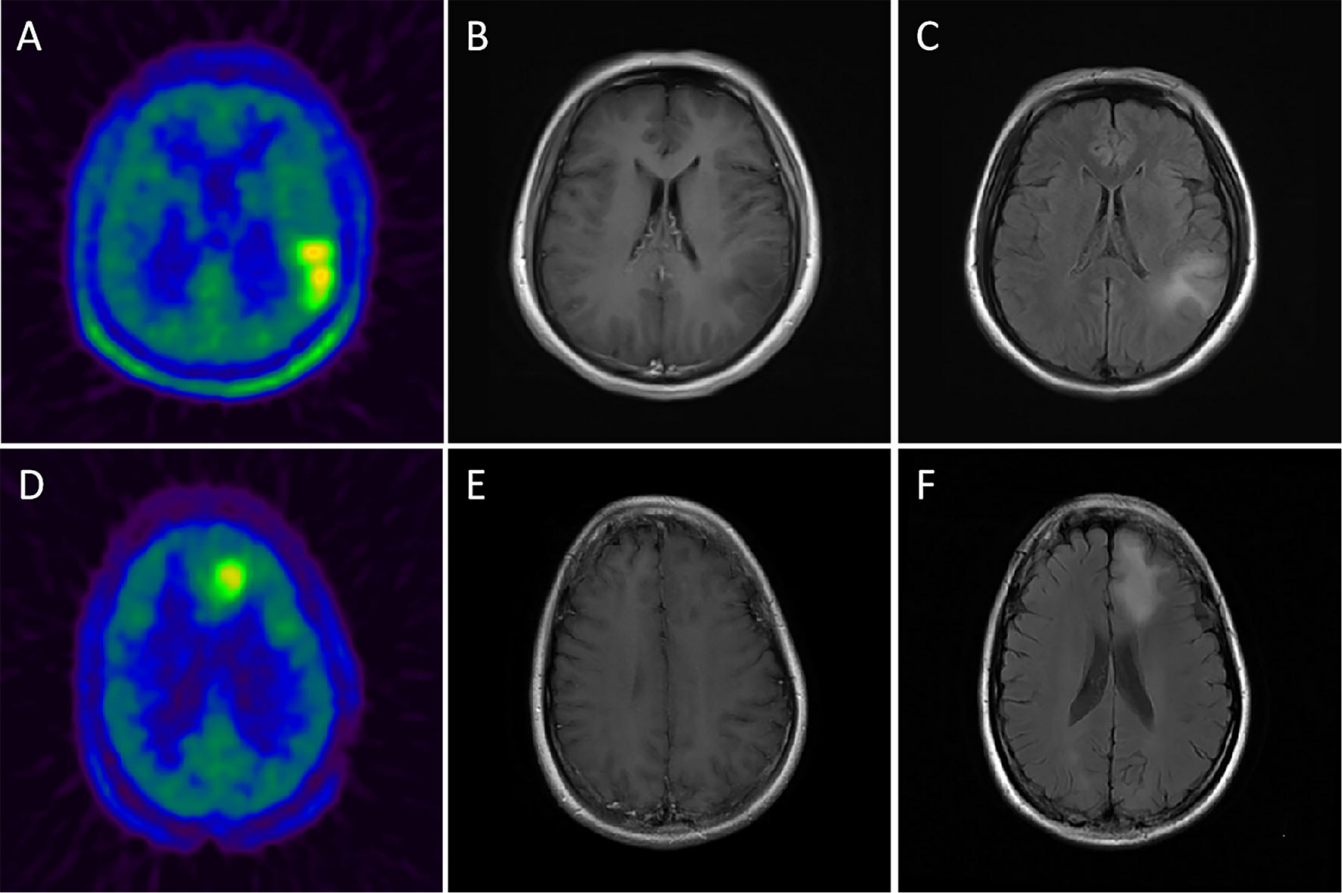
Upper row: example of a patient with a ^11^C-MET-positive lesion in the left temporo-parietal lobe, moderate TBRmax of 3.21 and moderate MTV of 46.1 ml **(A)** without contrast-enhancement in CE-T1 MRI **(B)** and an obvious flair alteration in T2 MRI **(C)** but with a moderate SUV_SD_ of 0.31 (>0.29); histopathological analysis revealed an IDH-wildtype, diffuse astrocytoma (WHO grade II). Lower row: example of a patient with a ^11^C-MET-positive lesion in the left frontal lobe, moderate TBRmax of 2.46 and moderate MTV of 31.3 ml **(D)** without contrast-enhancement in CE-T1 MRI **(E)** and a flair alteration in T2 MRI **(F)** but with a small SUV_SD_ of 0.26 (<0.29); histopathological analysis revealed an IDH-mutant, diffuse astrocytoma (WHO grade II).

After that, a nomogram was constructed on the basis of the multivariate logistic regression (for details, see Figure 1A). The nomogram (model 1) showed good discrimination efficacy, with a C-statistics of 0.866 (95%CI: 0.796–0.937). The calibration curve of the nomogram also indicated good agreement between predicted probability and actual occurrence in the whole cohort (Figure 1B). The Hosmer-Lemeshow test indicated no significant difference (*p* > 0.05), suggesting that there was no departure from a perfect fit. Meanwhile, we built model 2 only using MET PET information, i.e., brain midline structure involvement and SUV_SD_ derived from lesion VOI, with a C-statistics of 0.843 (95%CI: 0.766–0.920), suggesting that age information dichotomized by 45 years old do enhance the predictive ability for IDH genotype. Figure 2 illustrates a comparison of two representative grade II glioma cases with similar images by visual analysis. Our ^11^C-MET PET/CT-based nomogram could effectively distinguish between IDH-mutant and IDH-wildtype gliomas.

## Discussion

In the present study, we confirmed an association between volume-based ^11^C-MET PET quantification metrics and IDH mutational status for untreated glioma patients and further constructed a novel and intuitive statistical model to help clinicians and radiologists non-invasively predict glioma IDH mutation. As expected, our data demonstrated that TNRs and SUV_SD_ were significantly lower in the IDH-mutant group compared with those IDH-wildtypes, which are consistent with those of Kim et al. (15) ^11^C-MET PET derived SUV_SD_ showed the most excellent ability to identify whether glioma had an IDH mutation or not besides other MET PET metrics. Single-parameter SUV_SD_, which is a sort of tumor imaging heterogeneity feature, had the best prediction efficacy in IDH mutation. It is reasonable to hypothesize that the more heterogeneous the tumor MET PET imaging, the more likely IDH status is to be wildtype.

^11^C-MET PET played a significant role in evaluating the O_6_-methylguanylmethyltransferase methylation (MGMT) status in gliomas (27, 28). PET imaging was suggested to be informative for preoperatively differentiating gliomas according to 2016 WHO classification, particularly for differentiating IDH-wildtype and IDH-mutant tumors (19). A study of hybrid ^11^C-MET PET/MRI imaging including 39 glioma patients described that ROC analysis of TNRmax had a high AUC of 0.79 for predicting IDH status (16). Another study retrospectively evaluated 109 patients with newly diagnosed glioma also indicated that ^11^C-MET uptake was negatively correlated with IDH mutational status. The MET uptake of IDH-wildtype glioblastoma was significantly higher than that of IDH-mutant glioma (17). TNRmax derived from ^11^C-MET PET appears to be superior to MRS in differentiating IDH status with a ROC of 0.67 (18). The investigations on the relationship between amino acid tracer uptake and IDH status were not totally consistent. One O-(2-^18^F-fluoroethyl)-L-tyrosine(^18^F-FET) PET research in a mixed group of glioma patients, which included 16 oligodendrogliomas (IDH mutated and 1p/19q co-deleted), 27 astrocytomas (IDH mutated only) and 47 glioblastomas (IDH-wildtype), suggested that gliomas with IDH mutation are typically shown with a lower tumor to brain ratios(TNRmean and TNRmax), prolonged time to peak, and a slow-rise time-activity curve of 20–50 min (29). By contrast, another 3,4-dihydroxy-6-[^18^F]fluoro-L-phenylalanine (^18^F-FDOPA) imaging study in a total of 43 newly diagnosed glioma cases described paradoxically higher ^18^F-FDOPA uptake in diffuse grade II and III gliomas with IDH mutation (30). This inconsistency may be explained by the different amino acids PET probe uptake models in glioma (31). The expression level of L-type amino acid transporter in glioma is positively proportional to the intake value of MET, while the expression level of amino acid transporter is positively correlated with the microvascular density of glioma (32). Literature has shown that local blood flow in IDH wildtype glioma is higher than that of IDH mutants (33).

Our study differs from the abovementioned ones in that it indicated one predictive model for proper pre-operative prediction of IDH status in glioma patients. We aimed to develop a nomogram which is independent of histopathologic features, such as tumor grade or oligodendrocyte component, etc. The model showed good discrimination and was well-calibrated. Pre-operative lesion VOI SUV_SD_ should be important in daily clinical practice, which is a convenient and repetitive PET imaging parameter obtained through glioma VOI delineation. The origin of this PET imaging parameter could reflect the intratumoral heterogeneity to some degree. The SUV_SD_ difference derived from ^11^C-MET PET images between IDH-mutant and IDH-wildtype gliomas may help understand the possible internal link of intratumor heterogeneity and IDH mutation. Our model showed that the middle line structure involvement is associated with IDH mutational status. Things that need to be clarified is that this kind of brain midline structure involvement was also decided by VOI delineation, which could be more extensive and broader than the enhanced tumor volume in MRI. Moreover, age information has shown reasonable predictive potential and enhanced the predictive ability for IDH genotype. We report for the first time the application of ^11^C-MET PET/CT metrics and clinical age feature based nomogram in IDH genotyping for untreated glioma patients.

From specific clinical perspective, this nomogram model has some positive features. Firstly, our predictive model takes the advantage of being able to be rapidly acquired by a radiologist without requiring specialized software extracting texture features from high-order matrixes. Secondly, it is based on repetitive MET-PET metrics and some important clinical features, which is easily for understanding and clinically viable. This nomogram model displayed the potential to be used as a standalone diagnostic modality for patients with excessive surgical risk related to patient's comorbidities, advanced age, deep-seated, or brain stem tumors, etc.

There are some limitations to the present study. First, as this was a single-center study, with more cases are recruited, the training and validation group will be set for further external validation or multicenter validation to assess the potential clinical utility of our model further. Furthermore, next-generation sequencing for the IDH genotype was not available for this retrospective study, and some patients with the mutation may have been misidentified. Non-canonical IDH mutations can be found in IDH1 R132H immune-negative LGG (34). These points would be addressed in future work.

## Conclusions

This study proved that SUV_SD_ derived from regular glioma VOI delineation in MET PET imaging is a novel and convenient semiquantitative parameter for the glioma IDH prediction. The nomogram model combining with age, brain midline structure involvement, and SUV_SD_ demonstrates the potential in non-invasive IDH mutation status prediction for untreated glioma patients and showed reasonable convenience in clinical practice.

## Data Availability Statement

All datasets presented in this study are included in the article/Supplementary Material.

## Ethics Statement

All procedures performed in studies involving human participants were in accordance with the ethical standards of the institutional and national research committee (Ethics Committee of HuaShan Hospital Fudan University-approval number 2008-82) and with the principles of the 1964 Declaration of Helsinki and its later amendments or comparable ethical standards. Informed consent was obtained from all individual participants included in the study.

## Author Contributions

All authors listed have made a substantial, direct and intellectual contribution to the work, and approved it for publication.

## Conflict of Interest

The authors declare that the research was conducted in the absence of any commercial or financial relationships that could be construed as a potential conflict of interest.
