# Supplementary figures and images for: A Nomogram Modeling 11C-MET PET/CT and Clinical Features in Glioma Helps Predict IDH Mutation
Source: Front Oncol. 2020 Jul 24;10:1200. doi: 10.3389/fonc.2020.01200 (PMC7396495; doi:10.3389/fonc.2020.01200)

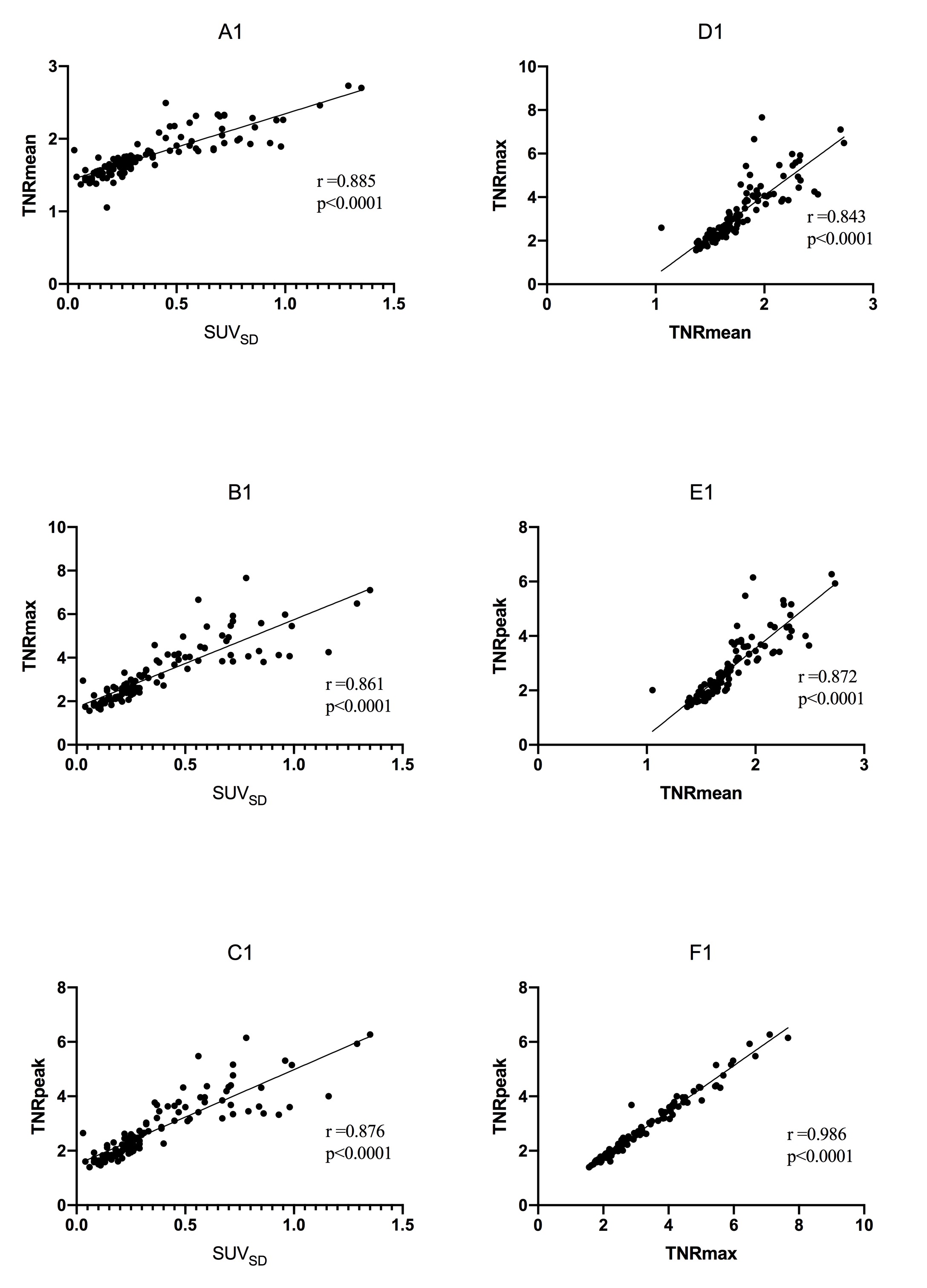

Supplement: Supplementary file 1 [file Image_1.JPEG]

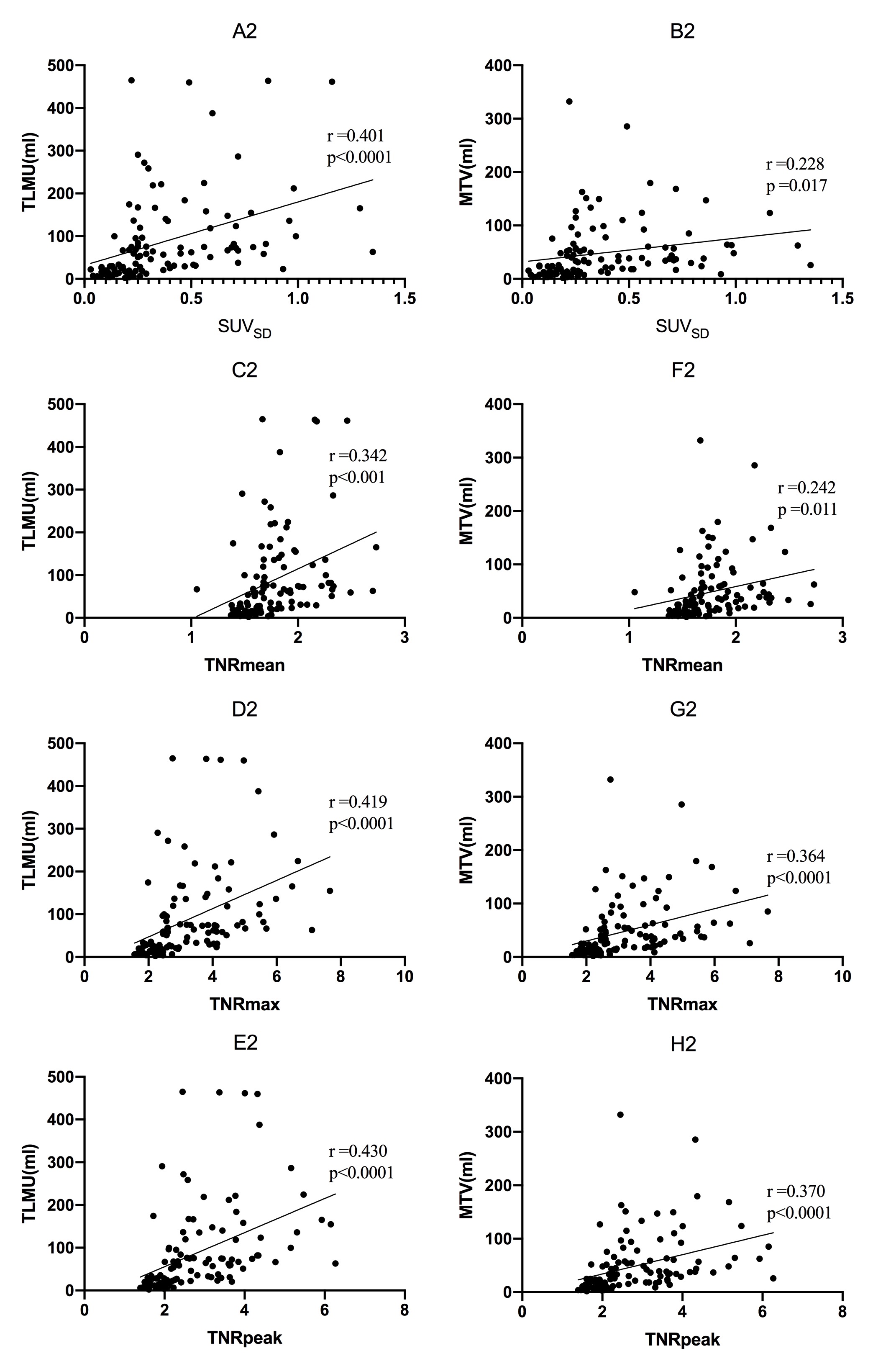

Supplement: Supplementary file 2 [file Image_2.JPEG]

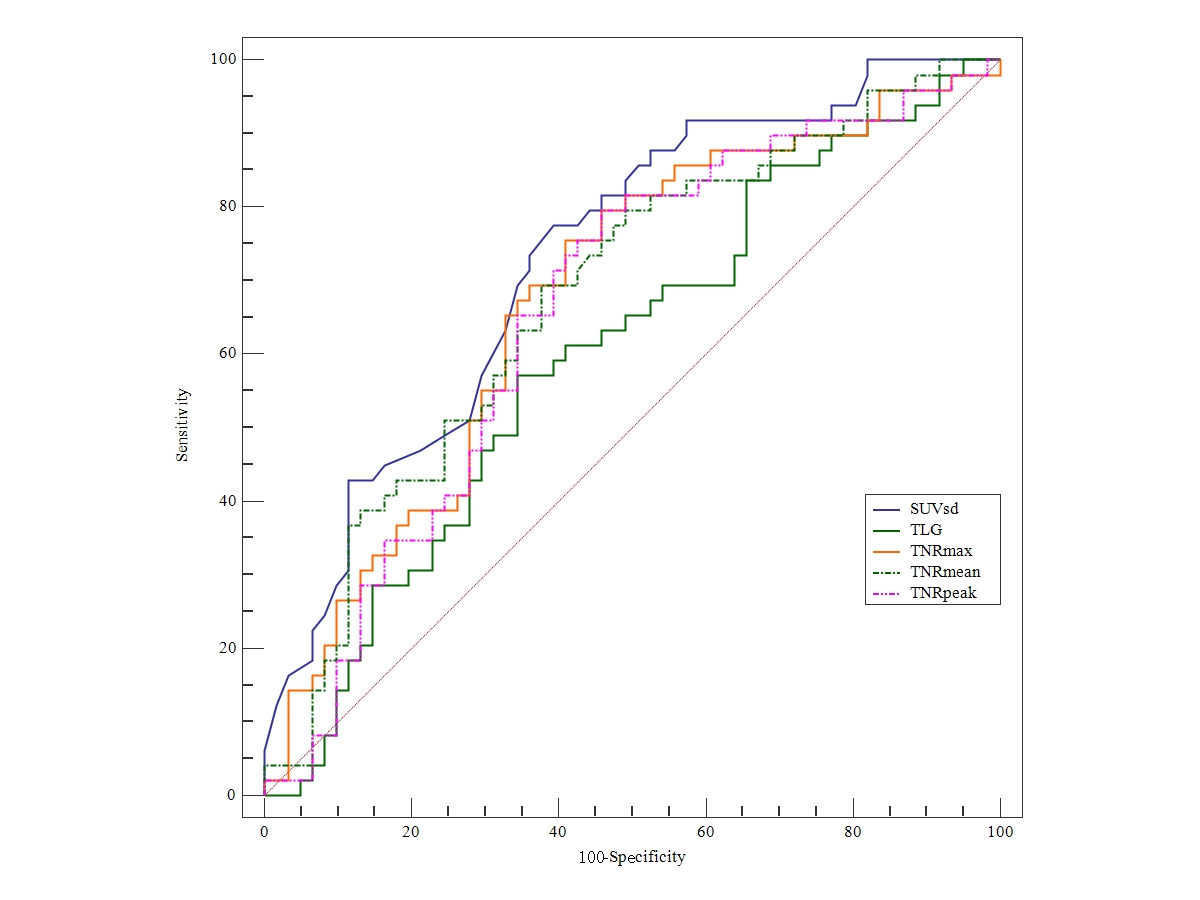

Supplement: Supplementary file 3 [file Image_3.JPEG]

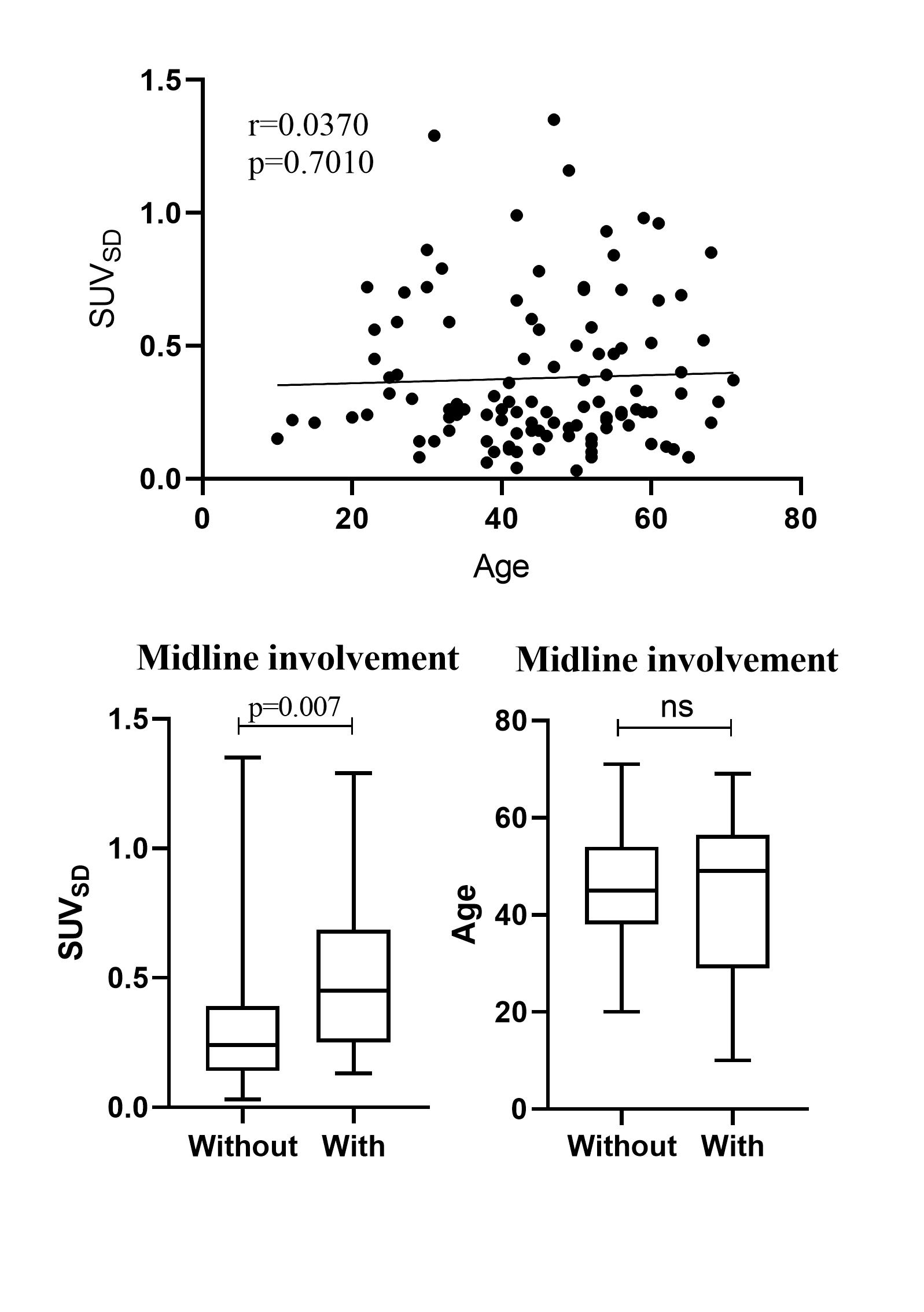

Supplement: Supplementary file 4 [file Image_4.JPEG]
