# Supplementary material for: A Nomogram Modeling 11C-MET PET/CT and Clinical Features in Glioma Helps Predict IDH Mutation
Source: Front Oncol. 2020 Jul 24;10:1200. doi: 10.3389/fonc.2020.01200 (PMC7396495; doi:10.3389/fonc.2020.01200)
Supplement: Supplementary file 5 [file Table_1.docx]

**Supplementary Table 1**：Pairwise comparison among ^11^C-MET PET metrics

| **Pairwise PET Metrics** | △AUC | Standard Error^a^ | Z-statistics | P value |
| --- | --- | --- | --- | --- |
| SUV_SD_ vs TNRmax | 0.0530 | 0.0222 | 2.384 | 0.0171 |
| SUV_SD_ vs TNRmean | 0.0522 | 0.0255 | 2.048 | 0.0406 |
| SUV_SD_ vs TNRpeak | 0.0708 | 0.0214 | 3.312 | 0.0009 |
| TNRmax vs TNRmean | 0.0009 | 0.0210 | 0.0399 | 0.9682 |
| TNRmax vs TNRpeak | 0.0177 | 0.0090 | 1.981 | 0.0475 |
| TNRmean vs TNRpeak | 0.0186 | 0.0199 | 0.935 | 0.3496 |
